# Second Harmonic Generation in Modal Phase-Matched Thin-Film Lithium Tantalate Ridge Waveguide

**DOI:** 10.3390/mi17050551

**Published:** 2026-04-29

**Authors:** Xiuquan Zhang, Haoyang Du, Dawei Cao, Jialu Duan, Qian Wang, Zhenyu Li, Wen Hu, Guiyin Liu, Lei Wang

**Affiliations:** 1 Key Laboratory of Laser and Infrared System, Ministry of Education, Shandong University, Qingdao 266000, China; zhangxiuquan@mail.sdu.edu.cn; 2 School of Physics, State Key Laboratory of Crystal Materials, Shandong University, Jinan 250100, China; haoyang.du@mail.sdu.edu.cn (H.D.); 2024cdw@mail.sdu.edu.cn (D.C.); duanjialu@mail.sdu.edu.cn (J.D.); qianwangsdu@mail.sdu.edu.cn (Q.W.); zhyli2021@mail.sdu.edu.cn (Z.L.); 3 Jinan Jingzheng Electronics Co., Ltd., Jinan 250100, China; huwen@nanoln.com (W.H.); liuguiyin@nanoln.com (G.L.)

**Keywords:** thin-film lithium tantalate, modal phase matching, second harmonic generation, integrated photonics

## Abstract

We demonstrate efficient and thermally stable second-harmonic generation (SHG) in *x*-cut thin-film lithium tantalate (TFLT) ridge waveguides via modal phase matching (MPM). The experimental characterizations reveal a normalized conversion efficiency (NCE) of 17.2% W^−1^ cm^−2^ in a 4 mm long waveguide. Notably, the device exhibits a temperature-dependent phase-matching wavelength slope of 0.007 nm/°C, which shows a two-orders-of-magnitude improvement in thermal stability over conventional periodically poled lithium niobate/lithium tantalate optical devices. Our work indicates that MPM in TFLT is an attractive strategy for integrated nonlinear optical applications, particularly for the on-chip frequency conversion of both classical and quantum light signals without on-chip domain-poling processes.

## 1. Introduction

Nonlinear optical (NLO) phenomena governed by second-order susceptibility (*χ*^(2)^) underpin a wide range of critical photonic functions, such as second-harmonic generation (SHG) [1], sum- and difference-frequency generation (SFG/DFG) [2], optical parametric amplification and oscillation (OPA/OPO) [3,4,5,6], supercontinuum generation (SCG) [7,8], and spontaneous parametric down-conversion (SPDC) [9,10]. Among these processes, SHG is critically important, serving as the essential precursor that validates material quality, phase-matching design, and device fabrication—while simultaneously enabling direct applications in wavelength conversion and providing a building block for higher-order nonlinear interactions [11].

The emergence of thin-film lithium tantalate (TFLT, lithium tantalate on insulator, LTOI) has positioned it as an attractive platform in integrated nonlinear photonics. TFLT enables waveguide structures with high-refractive-index contrasts, supporting sub-micron guiding modes and compact bending structures (with radii of tens of microns), thereby facilitating the development of high-performance nanophotonic devices [12,13,14]. TFLT has several excellent optical properties, including a broad transparency window (0.28–5.5 µm) [15], a low birefringence of 0.004 [13], a low microwave loss tangent of 6.5 × 10^−4^ [16], and a high threshold for the photorefractive effect [17]. Recent advances in high-quality TFLT have created new opportunities for developing high-performance integrated photonic platforms [13,18,19].

For efficient second-harmonic generation (SHG), phase matching is critical. The most widely investigated techniques for achieving phase matching are quasi-phase matching (QPM) [20,21,22], birefringent phase matching (BPM) [23] and modal phase matching (MPM) [24,25]. Several SHG devices using the QPM process based on periodically poled TFLT (PP-TFLT) have already been developed, demonstrating a normalized conversion efficiency (NCE) exceeding 200% W^−1^ cm^−2^, which showcases the promise of this platform for nonlinear photonic applications [26,27]. However, the QPM process is susceptible to waveguide dimensions, poling periods, and duty cycles, requiring careful experimental calibrations/considerations to ensure the reproducibility of the phase-matching wavelength and conversion efficiency [28,29]. BPM matches refractive indices via angle and polarization, enabling applications in thin-film lithium niobate (TFLN) [30], which experimentally demonstrate an NCE of 2.7% W^−1^ cm^−2^ with a thermal tuning slope of 1.06 nm/°C. However, spatial walk-off reduces interaction length and efficiency. In the TFLT platform, the low birefringence makes BPM particularly challenging.

MPM avoids the need for complex periodic poling and has shown great potential in integrated nonlinear photonics [31]. Although theoretical investigations of MPM-based SHG on the TFLT platform have been reported recently [32], few experimental realizations have been demonstrated. Therefore, a quantitative understanding of the relationship between waveguide geometry, phase-matching wavelength, and thermal tuning behavior is essential for reproducible device engineering. In this work, we demonstrate an efficient and thermally stable SHG on *x*-cut TFLT ridge waveguides by using MPM. The waveguide design enables phase matching between the fundamental TE_00_ mode of the pump and the high-order TE_20_ mode of the SH signal. Our investigations show an efficient SHG in fabricated devices, achieving an NCE of 17.2% W^−1^ cm^−2^ in a 4 mm long waveguide. The devices exhibit an exceptional thermal stability, with d*λ_PM_*/d*T* = 0.007 nm/°C, representing a substantial improvement over the former investigations.

## 2. Simulation and Fabrication Details

**Figure 1:**
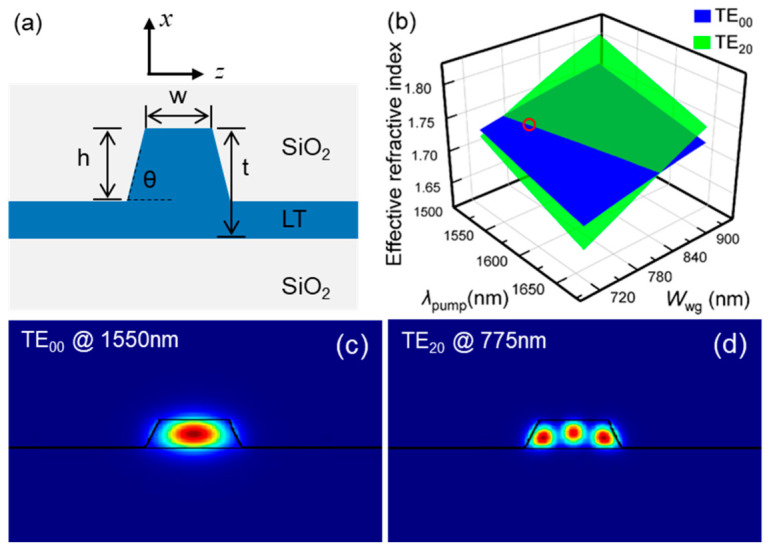
Design and MPM simulation results of a 410 nm thick *x*-cut TFLT ridge waveguide. (**a**) Cross-sectional view of the ridge waveguide. (**b**) Simulation results of the effective refractive indices (*n*_eff_) of the fundamental TE_00_ mode (blue landscape) and the high-order TE_20_ mode (green landscape) as functions of the variations in pump wavelength *λ*_Pump_ and waveguide top width *W*_wg_. (**c**,**d**) The simulated mode profiles for TE_00_ mode and TE_20_ mode at 1550 nm and 775 nm, respectively.

Our approach relies on MPM between both quasi-transverse-electric (qTE) modes: the fundamental TE_00_ mode at pump light *λ*_Pump_ and the high-order TE_20_ mode at second-harmonic (SH) light *λ*_SH_. The TE_10_ mode, despite being a higher-order mode, has an anti-symmetric field distribution. As a result, its overlap integral with the fundamental TE_00_ pump mode is nearly zero, which makes it ineffective for efficient SHG. Hence, the TE_20_ mode is the lowest-order qTE mode that yields a non-zero modal overlap. Figure 1a illustrates the design of the TFLT ridge waveguide device. Figure 1b presents the simulation results of the effective refractive index (*n*_eff_) of TE_00_ (blue landscape) and TE_20_ (green landscape) assuming a film thickness of 410 nm. The intersection of these two landscapes indicates the phase-matching condition *n*_eff_ (*λ*_Pump_) = *n*_eff_ (*λ*_SH_). By fixing the pump wavelength at 1550 nm, the waveguide top width *W*_wg_ ≈ 770 nm is determined according to the phase-matching condition, as marked by the red circle in Figure 1b. The mode profiles of TE_00_ at 1500 nm and TE_20_ at 775 nm, with electric fields dominantly confined to the *xz*-plane, are shown in Figure 1c and Figure 1d, respectively. The theoretical NCE (*η*_nor_) of SHG can be calculated using the following expression [33]:

(1) $$\eta_{nor}\equiv \frac{8\pi^{2}}{\epsilon_{0}cn_{1}^{2}n_{2}\lambda^{2}}\frac{\zeta^{2}d_{eff}^{2}}{A_{eff}}$$

Here, *d*_eff_ represents the effective nonlinear susceptibility, *λ* is the pump wavelength, and *ϵ*_0_ and *c* are the permittivity and speed of light in a vacuum, respectively. *n*_1_ and *n*_2_ are the *n*_eff_ of the pump mode and SH mode, respectively. In this equation, $A_{eff}={\left(A_{1}^{2}A_{2}\right)}^{\frac{1}{3}}$ is the effective mode area, where $A_{i}=\frac{{(\int }_{all}|{\overset{\to }{E}}_{i}{|}^{2}dxdz{)}^{3}}{|\int_{\chi^{\left(2\right)}}|{\overset{\to }{E}}_{i}{|}^{2}{\overset{\to }{E}}_{i}dxdz{|}^{2}},(i=1,2)$, while *A*_1_ and *A*_2_ are the pump mode area and SH mode area, respectively, and *ζ* represents the spatial mode overlap factor between the pump and SH waves, given as

(2) $$\zeta =\frac{\int_{\chi^{\left(2\right)}}{\left(E_{1z}^{*}\right)}^{2}E_{2z}dxdz}{|\int_{\chi^{\left(2\right)}}|{\overset{\to }{E}}_{1}{|}^{2}{\overset{\to }{E}}_{1}dxdz{|}^{\frac{2}{3}}|\int_{\chi^{\left(2\right)}}|{\overset{\to }{E}}_{2}{|}^{2}{\overset{\to }{E}}_{2}dxdz{|}^{\frac{1}{3}}}$$

where $\int_{\chi^{\left(2\right)}}$ and $\int_{all}$ denote two-dimensional integrations over the *χ*^(2)^ material and the entire computational domain, respectively. ${\overset{\to }{E}}_{1}$ and ${\overset{\to }{E}}_{2}$ are the electric fields of the pump and SH waves, respectively, and *E*_1*z*_ and *E*_2*z*_ are their *z*-components. 

The above equations demonstrate that SHG efficiency fundamentally hinges on three key parameters: the spatial mode overlap factor *ζ*, the effective mode area *A*_eff_, and the effective nonlinear susceptibility *d*_eff_. Numerical simulations reveal that our waveguide achieves an effective mode area *A*_eff_ of 0.7 µm^2^ and a spatial mode overlap *ζ* of 0.08. Consequently, with an effective nonlinear susceptibility *d*_eff_ of 13.8 pm/V, the device demonstrates an NCE reaching 33.9% W^−1^ cm^−2^.

**Figure 2:**
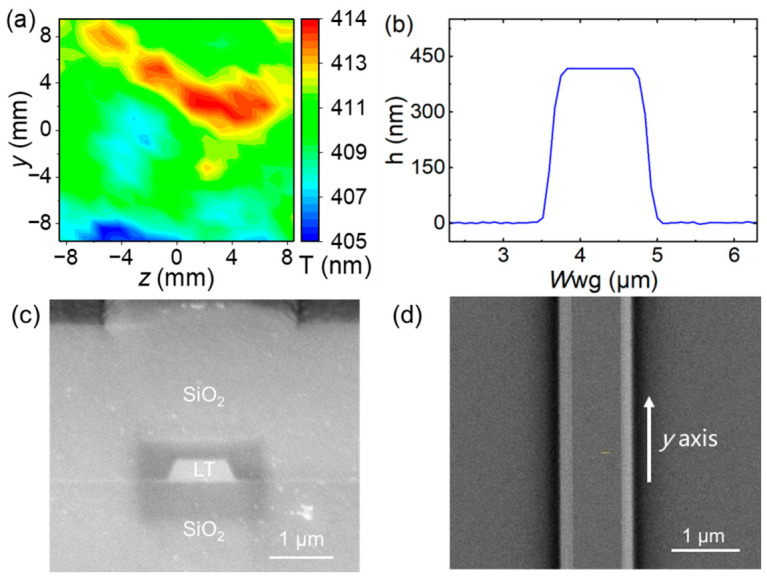
TFLT ridge waveguide fabrication details. (**a**) Thickness range of the TFLT film in the device region characterized using interferometer (Filmetrics F50). (**b**) Cross-sectional view of the waveguide observed by AFM before SiO_2_ cladding, showing the waveguide top width 859 nm, the height 400 nm, and the sidewall angle 67°. *W*wg, waveguide top width; h, waveguide height. (**c**,**d**) SEM images of the waveguide from the top view (before SiO_2_ cladding) and cross-section view (after SiO_2_ cladding), respectively.

The devices were fabricated by using standard e-beam lithography (EBL) and Ar^+^ ion-beam etching on an *x*-cut TFLT wafer (from NANOLN, Jinan, China) with an average thickness of 410 nm. The thickness of the buried SiO_2_ layer is about 4.7 µm. To validate the refractive index model employed in our phase-matching simulations (Figure 1b), we characterized both the thickness uniformity and the refractive index of the TFLT film in the device region. Interferometer measurements (Filmetrics F50, KLA Instruments, Milpitas, CA, USA) reveal a thickness variation of less than ±2.5 nm across the entire device area (Figure 2a), which translates into a phase-matching wavelength shift of approximately 2 nm—well within the experimental resolution. Furthermore, prism-coupling measurements at 632.8 nm yield ordinary and extraordinary refractive indices of *n*ₒ = 2.1769 and *n*ₑ = 2.1814, respectively. Both values agree with the predictions of the Sellmeier equation for congruent LT to within 0.0005. These results collectively validate the refractive index model employed in our simulations. Given the high sensitivity of the MPM wavelength to waveguide geometric parameters, a series of waveguides with varying top widths (increments ~20 nm) around 770 nm were fabricated. After the etching process, the devices were coated with a 3 µm thick SiO_2_ layer prepared using plasma-enhanced chemical vapor deposition (PECVD). Figure 2 presents the morphological characterization of the fabricated waveguide with a top width of 859 nm, including AFM cross-sections (Figure 2b) and SEM images (Figure 2c,d), confirming the high structural fidelity of the ridge waveguides. Following end-face polishing, the ridge waveguide devices were subjected to the characterization process.

**Figure 3:**
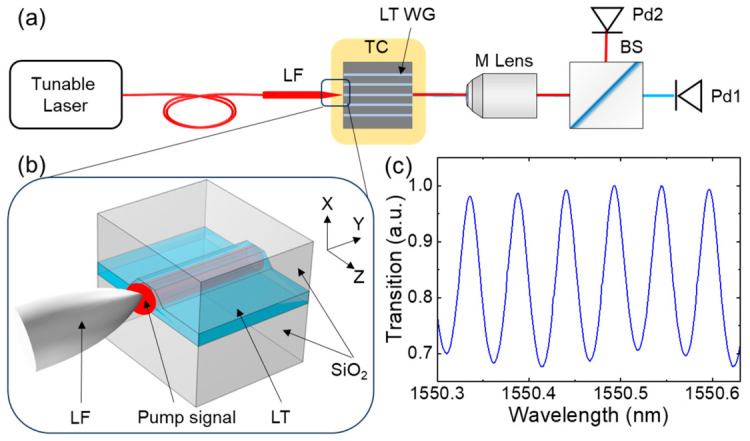
Schematic of the SHG experimental setup (**a**) and schematic of pump signal coupling from a lensed fiber into a waveguide (**b**). Tunable Laser, TSL-570C; LF, lensed fiber; LT WG, LT waveguide; TC, temperature controller; M Lens, microscope lens; BS, beam splitter; Pd1 and Pd2, InGaAs photodetector and Si photodetector. (**c**) The Fabry–Perot interference fringe of the waveguide with a length of 11 mm.

The experimental setup for SHG characterization is depicted in Figure 3a,b. A tunable laser (TSL-570C, Santec, Komaki, Japan) operating in the telecom band served as the pump source, coupled into the waveguide via a lensed fiber. The output light was separated using a beam splitter and directed to the appropriate photodetectors for measurement. Precise temperature control was maintained using a temperature controller to investigate thermal stability characteristics. The pump wavelength is scanned from 1500 nm to 1630 nm while monitoring the generated near-infrared light to obtain the SHG spectrum. The Fabry–Perot interference method was employed to measure waveguide propagation loss in the telecom band. Observed intensity fringes (Figure 3c) yielded a propagation loss of 1.3 dB/cm [34]. We also fabricated microring resonators and characterized the losses, as detailed in the Appendix A.1.

## 3. Results and Discussion

### 3.1. SHG and Conversion Efficiency

**Figure 4:**
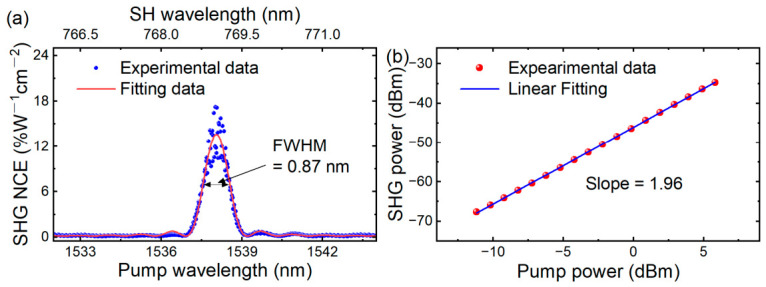
SHG from a TFLT ridge waveguide with a length of 4 mm and a top width of 760 nm. (**a**) Conversion efficiency spectrum with the center wavelength of the sinc^2^-function aligned to the measured peak. (**b**) SH power as a function of pump power, with experimental data compared with a quadratic fitting.

For the modal phase-matched waveguide with a top width of 760 nm, the SHG peak occurred at a pump wavelength of *λ*_Pump_ = 1538 nm, corresponding to an SH wavelength of *λ*_SH_ = 769 nm. Figure 4a shows the wavelength-dependent SHG signal in a 4 mm long waveguide, confirming the presence of a phase-matching wavelength. We measured an SH power of 20.6 nW under a pump power of 590.3 µW. The combined transmission efficiency of the objective lens and beam splitter was measured at 63.6% for the pump wavelength (1538 nm) and 86.8% for the SH wavelength (769 nm), determined via a reference measurement conducted in the absence of the waveguide in the optical path. The overall fiber-to-fiber loss of the setup was 10.3 dB. Accounting for a propagation loss of 1.3 dB/cm, the extracted fiber-to-chip coupling loss is 4.9 dB/facet. By calibrating out the losses of optical components on both the pump light and the SH light, we estimated an NCE of 17.2% W^−1^ cm^−2^ ($\eta_{nor}=\frac{P_{SH}}{P_{pump}^{2}L^{2}}$). The main lobe of the recorded conversion efficiency spectrum agrees well with the theoretical sinc^2^(Δ*βL*/2) function, exhibiting a fitted SHG efficiency of 13.6% W^−1^ cm^−2^ and a 3 dB bandwidth of 0.87 nm. The propagation loss of the SH wave is estimated to be ~5.2 dB/cm based on that of the pump wave [33]. Accounting for both the pump and SH wave propagation losses, the effective interaction length *L*_eff_ is calculated to be 1.16 cm [35], which exceeds our device length, and the calculated NCE is estimated to exceed 27% W^−1^ cm^−2^.

Experimental conversion efficiency (17.2% W^−1^ cm^−2^) falls below simulation predictions (33.9% W^−1^ cm^−2^). This is primarily due to fabrication-induced geometrical deviations and the propagation loss. Specifically, a quantitative sensitivity analysis reveals that a dimensional deviation of ±10 nm in waveguide thickness can significantly alter the mode profiles, reducing the spatial mode overlap factor ζ by approximately 5%, as detailed in Appendix A.4. This accounts for the differences between theoretical and measured NCE values. However, the agreement between theoretical calculation and the measured SHG curve confirms the SHG characteristics of the TFLT device. Further optimization of fabrication processes, particularly through chemical–mechanical polishing (CMP) to improve surface smoothness and waveguide uniformity, is expected to improve the conversion efficiency in the following implementations [36]. To verify the dependence of SH power on the pump signal power, we conducted SHG experiments at various pump power levels. The pump wavelength was fixed within a very narrow range (1537.5–1538.5 nm) at the wavelength corresponding to the peak SHG conversion efficiency. Different pump signal power levels were applied, and then the SH power was recorded for each pump power. Limited by the output power of our tunable laser and the insertion loss of our device, the pump power inside the waveguide was approximately 3500 µW. No photorefractive damage or optical instability were observed during the measurement. Details about the maximum measured pump power and SH power can be found in Appendix A.2. Power-dependent measurements yield a fitted slope of 1.96 (Figure 4b), confirming the quadratic relationship between the SH power and the pump power, consistent with theoretical expectations according to the equation described by $\eta_{nor}\eta =\frac{P_{SH}}{P_{pump}^{2}}$. Through fitting the experimental data, an on-chip conversion efficiency of 2.75% W^−1^ is obtained.

### 3.2. Thermal Stability of the Phase-Matching Wavelength

**Figure 5:**
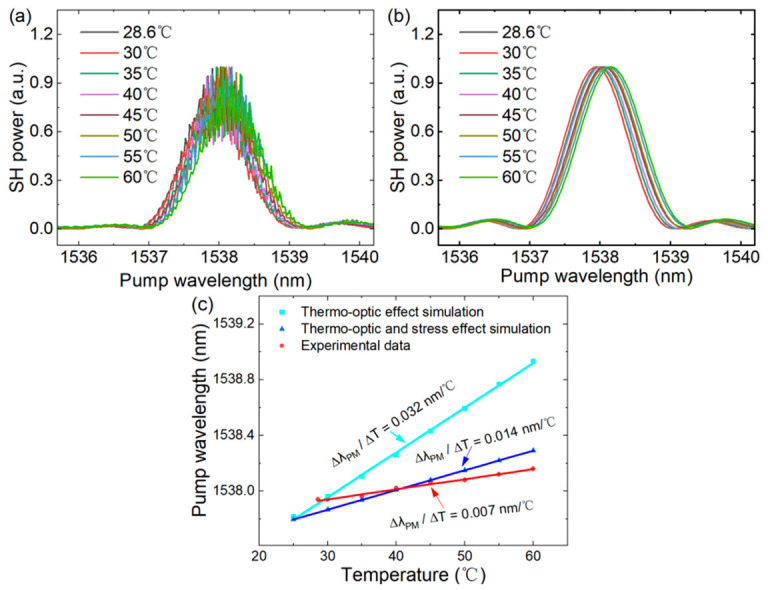
Thermal tuning of SHG. (**a**,**b**) Conversion efficiency spectra at different temperatures. Each spectrum is normalized by its peak value for comparison. (**a**) Experimental data. (**b**) Fitting data. (**c**) Simulation and experimental linear fitting, showing the thermal tuning rates d*λ_PM_*/d*T* of 0.032 nm/°C (simulation of thermo-optic effect on thermal tuning rate), 0.014 nm/°C (simulation of thermo-optic and thermal stress-induced effect on thermal tuning rate) and 0.007 nm/°C (experimental data), respectively.

The thermal stability characteristics were investigated by varying the device temperature from 28.6 °C to 60 °C. Figure 5a,b plot the measured SH spectra and fitting curve at different temperatures and the corresponding phase-matching pump wavelengths *λ*_Pump_, respectively. Linear fitting yields a thermal tuning rate of d*λ*_PM_/d*T* = 0.007 nm/°C, as shown by the red line in Figure 5c. We summarized the reported thermal tuning rate results in Table 1 for a quick examination. Compared with the temperature-dependent phase-matching wavelength shift of 0.44 nm/°C demonstrated in the PP-TFLT device [26], our work exhibits near-zero thermal drift, which is critical for applications demanding stringent temperature stability and precision, such as quantum information processing [37].

**Figure A5:**
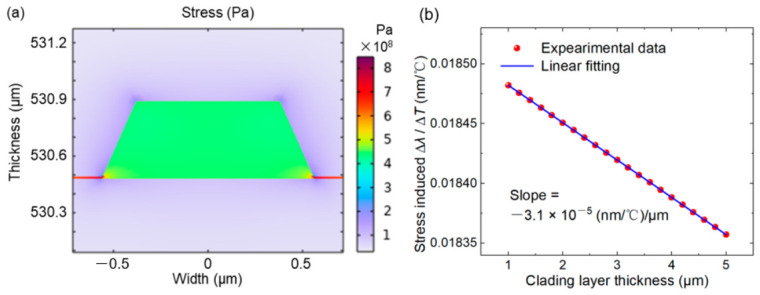
Simulation of thermal stress-induced thermal tuning rate. (**a**) Stress distribution induced by thermal expansion mismatch among the TFLT and SiO_2_ cladding. (**b**) Linear fitting of thermal tuning rate affected by SiO_2_ cladding thickness. Linear fitting result indicates that increasing the cladding thickness from 1 to 5 µm changes the stress-induced tuning rate by about −1.24 × 10^−4^ nm/°C.

The exceptional thermal stability of the TFLT waveguide stems from the synergistic combination of its inherent material properties, the MPM mechanism, and the SiO_2_-cladding configuration. The thermal stability of the present TFLT device was examined by numerically simulating the phase-matching wavelength as a function of temperature, neglecting the thermal expansion mismatch between the SiO_2_ cladding layer (~0.5 × 10^−6^/°C) and the LT film (~1.6 × 10^−6^/°C) [41,42]. The calculated results are presented in Figure 5c, showing a thermal tuning rate of 0.032 nm/°C. A large deviation between the numerical simulations and experimental measurements suggests that the present thermo-optic coefficient-based model is not suitable for the present device. Thermal expansion mismatch between the SiO_2_ buried layer and the TFLT ridge results in interfacial stress, leading to a stress-induced refractive index change in the TFLT device. A recent investigation suggests that the thermal tunability of the integrated platform can be changed by the SiO_2_-cladding layer [43]. The thermo-optic coefficient (TOC) of the on-chip device is determined using the following equation:

(3) $$\frac{d\lambda }{dT}=\lambda (\frac{dn_{eff}}{dT}-\frac{\nu }{E}\frac{d\sigma }{dT})$$

where *E* and *ν* are the Young modulus and the Poisson constant of the waveguide, respectively, and σ is the temperature-induced stress components caused by the thermal expansion coefficient (TEC) difference between the waveguide and the cladding material. The first term $\frac{dn_{eff}}{dT}$ represents the thermo-optic effect originating from the core material, while the second term $\frac{\nu }{E}\frac{d\sigma }{dT}$ accounts for thermal stress-induced drift. The experimental thermal tuning rate (0.007 nm/°C) can be explained by the stress-induced refractive index variation arising from the thermal expansion mismatch between the silica layer and the LT thin film, which effectively counteracts the intrinsic thermo-optic effect of TFLT. When the thermal stress is taken into account in our numerical simulations, the thermal tuning rate is found to be 0.014 nm/°C (see Appendix A, Figure A5b), which shows a better consistency with the experimental value. The slightly lower tuning slope obtained experimentally can be partially ascribed to pyroelectric and thermal expansion effects acting on the waveguide cross-section, which are absent in the simulation model [24]. The underlying governing physics will be elucidated in our future work.

### 3.3. Dependence of Phase-Matching Wavelength on Waveguide Top Width

**Figure 6:**
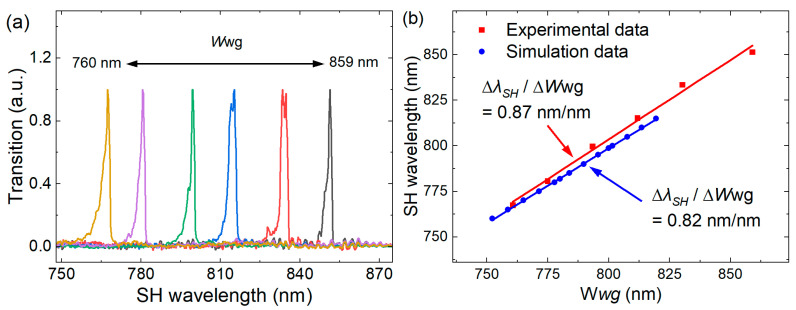
Wavelength-dependent SHG signals for waveguides with different top widths. (**a**) Conversion efficiency spectrum pumped by the supercontinuum laser (SC-5F) and detected by the optical spectrum analyzer (OSA, AQ6374, YOKOGAWA, Tokyo, Japan) with fiber–waveguide–fiber coupled. (**b**) SH wavelength as a function of top width *W*_wg_, with experimental data compared to a linear fitting.

We also investigate the influence of waveguide geometry on the pump wavelength by performing SHG experiments on six waveguides (length: 11 mm; top widths: 760–859 nm; SEM images of the six waveguide top widths can be found in Appendix A.3). Using a supercontinuum laser (wavelength range: 1400–1700 nm) as the pump source, the SHG spectrum beyond the wavelength range of TSL-570 was investigated. As shown in Figure 6a, even with a broad-spectrum laser source, only a single SHG peak is observed from each waveguide. Comparing the experimental results shown in Figure 4a and Figure 6a, the former reveals that an 11 mm long waveguide exhibits increasingly pronounced side lobes in the efficiency spectrum. This characteristic likely arises from minor nonuniformities in waveguide dimensions along the propagation direction, confirming that the phase-matching condition is highly sensitive to geometric parameters. We observe a linear relationship between phase-matching wavelength and waveguide top width, with an experimental slope of d*λ_SH_*/d*W*_wg_ = 0.87 nm/nm, which agrees well with the simulated value of 0.82 nm/nm. This consistency enables precise wavelength engineering for target applications. Over the 11 mm waveguide length, the side lobes of *λ*_SH_ are offset from the main peak by ~7 nm. Using the linear relationship d*λ*_SH_/dW_wg_ = 0.82 nm/nm, we estimate a waveguide width variation of about ±4.2 nm along the propagation direction. Therefore, optimizing the fabrication process to enhance the device uniformity is expected to further improve the conversion efficiency.

## 4. Conclusions

In conclusion, we demonstrate efficient and thermally stable SHG in *x*-cut TFLT ridge waveguides via MPM. The experimental NCE is about 17.2% W^−1^ cm^−2^ in a 4 mm long waveguide. The temperature-dependent measurements highlight the strong thermal robustness of TFLT (0.007 nm/°C), demonstrating approximately a two-orders-of-magnitude improvement over conventional periodically poled devices. Our results imply TFLT as a promising platform for integrated nonlinear photonics, especially for applications that require both high nonlinear efficiency and reliable operational stability.

## Figures and Tables

**Table 1:** Thermal tuning rate comparison of representative SHG devices.

Year	*λ*_PM_ (nm)	Thermal Tuning Rate	Efficiency (%W^−1^ cm^−2^)	Length	Type	References
2018	1550	0.065 nm/°C	26/61	—	MPM	[31]
2020	1535	−1.71 nm/°C	2400	1 mm	QPM	[38]
2021	1574.6	~0.137 nm/°C	5540/6891	1.2 mm	MPM	[25]
2022	1535.1	1.06 nm/°C	2.7/—	20.0 mm	BPM	[30]
2023	1550	1.01 nm/°C	2.7	20.0 mm	QPM	[39]
2023	1550	2.5 nm/°C	72.1 (predicted value)	—	MPM	[40]
2025	1568	−0.44 nm/◦C	229/	6.5 mm	QPM	[26]
2025	1552	—	208/244	12 mm	QPM	[27]

**Figure A1:**
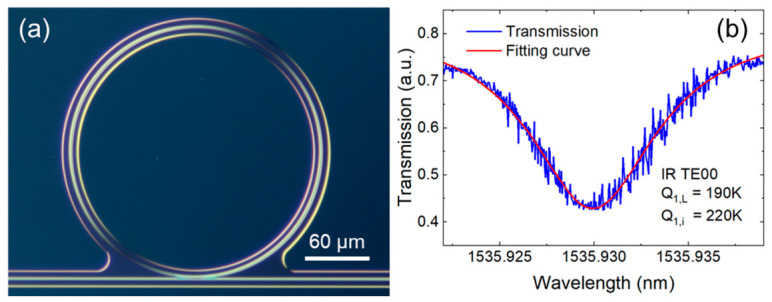
(**a**) Microscope picture of the microring resonator. (**b**) Transmission spectrum of a typical resonance, with experimental data shown in blue and a fitting curve shown in red.

**Figure A2:**
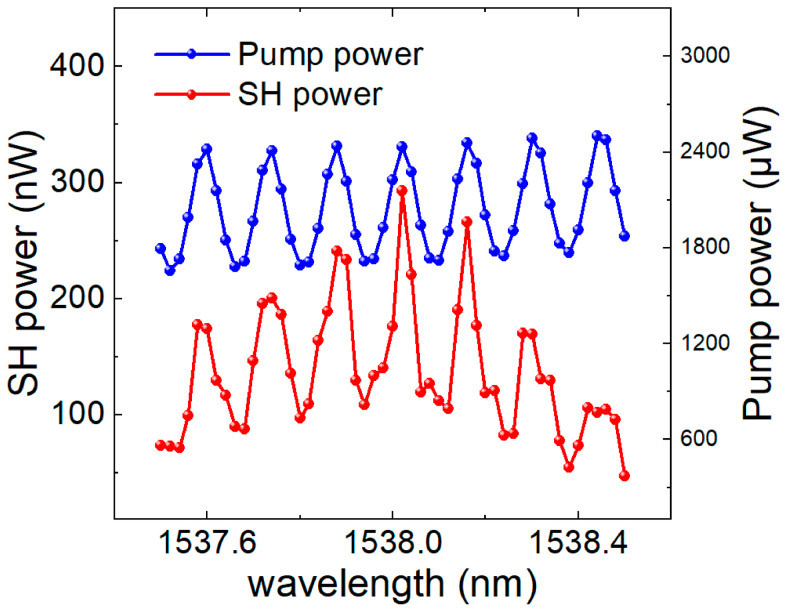
Experimental data of maximum pump power and SHG power.

**Figure A3:**
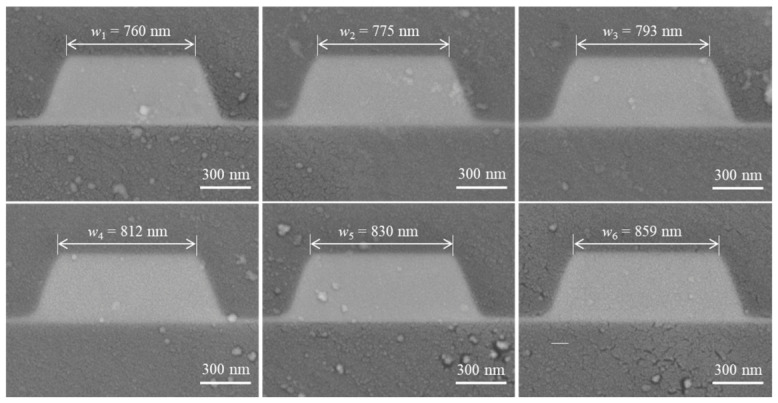
SEM images of top widths of the six waveguides.

**Figure A4:**
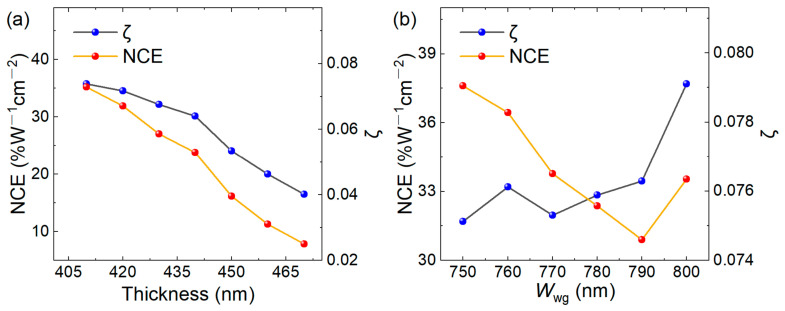
Sensitivity analysis of the mode overlap factor ζ and normalized conversion efficiency (NCE) to fabrication-induced variations in waveguide (**a**) thickness and (**b**) top width *W*_wg_.

## Data Availability

The original contributions presented in this study are included in the article. Further inquiries can be directed to the corresponding author.

## Appendix A

### Appendix A.1. Microring Resonator Transmission Loss Characterization

To obtain more precise propagation loss measurements, microring resonators with a radius of 100 µm (selected to minimize bending loss) were fabricated and characterized (Figure A1). The device is tested with an experimental setup similar to that shown in Figure A1a, except that the photodetector has been replaced with an oscilloscope to achieve finer step size detection of the emitted light. Figure A1b presents the detailed transmission spectrum of a typical resonance with an intrinsic Q of 2.2 × 10^5^, corresponding to a propagation loss of 1.8 dB/cm in the telecom band.

### Appendix A.2. Maximum SHG Power Measurement

Details of the maximum measured pump and SH power are shown in Figure A2. By calibrating out the losses of optical components such as the objective lens and beam splitter on both the pump light and the SH light, the maximum SH power is estimated to be 337.7 nW, corresponding to a pump power of about 3500 µW.

### Appendix A.3. SEM Images of the Waveguide Top Width

SEM images of the six waveguide top widths are shown below.

### Appendix A.4. Sensitivity of NCE to Waveguide Geometric Deviations

To understand the discrepancy between the simulated and measured NCE, we analyzed the sensitivity of the phase-matched waveguide to fabrication-induced waveguide geometrical deviations. Numerical simulation results show that the overlap factor ζ is highly sensitive to small variations in the waveguide dimensions near the optimal geometry. For example, for a waveguide with a thickness near 410 nm, a ±5 nm deviation in waveguide thickness reduces the ζ by more than 5% and the NCE by over 12%. Similarly, small top width deviations on the order of several nanometers also lead to a reduction in NCE. This sensitivity indicates that nanometer-scale fabrication deviations can significantly degrade the conversion efficiency and partially account for the difference between the simulated value (33.9% W^−1^ cm^−2^) and the measured value (17.2% W^−1^ cm^−2^).

### Appendix A.5. Thermo-Mechanical Analysis of Stress-Induced Thermal Tuning Rate

To address the concern of the thermal stress-induced thermal tuning rate, we have carried out additional thermo-mechanical simulations using COMSOL v6.4, to evaluate the stress distribution induced by thermal expansion mismatch among the TFLT and SiO_2_ cladding. The simulations were performed at a pump wavelength of 1550 nm, and the material parameters for SiO_2_ were set as Young’s modulus = 77 GPa and Poisson’s ratio = 0.21, which are typical values for PECVD-deposited SiO_2_. The simulation results show a stress level of ~100 MPa (Figure A5a) and a stress-induced thermal tuning rate of about 0.018 nm/°C (Figure A5b), which partially compensates for the intrinsic thermo-optic shift. Since the thermal expansion mismatch between the TFLT and the SiO_2_ cladding is a fundamental property of the material stack, the stress-induced compensation mechanism is inherently predictable and reproducible, rather than a random fabrication artifact. The tunability via PECVD parameters further supports its controllable nature [43].
